# MAG2, a Toxoplasma gondii Bradyzoite Stage-Specific Cyst Matrix Protein

**DOI:** 10.1128/mSphere.00100-20

**Published:** 2020-02-19

**Authors:** Vincent Tu, Joshua Mayoral, Rama R. Yakubu, Tadakimi Tomita, Tatsuki Sugi, Bing Han, Tere Williams, Yanfen Ma, Louis M. Weiss

**Affiliations:** a Department of Pathology, Albert Einstein College of Medicine, Bronx, New York, USA; b Department of Medicine, Albert Einstein College of Medicine, Bronx, New York, USA; Indiana University School of Medicine

**Keywords:** latency, monoclonal antibody screen, intravacuolar network, MAG1, MAG2, *Toxoplasma gondii*, bradyzoite, cyst matrix, fluorescence recovery after photobleaching (FRAP)

## Abstract

Toxoplasma gondii causes a chronic infection that affects a significant portion of the world’s population, and this latent infection is the source of reactivation of toxoplasmosis. An attribute of the slowly growing bradyzoite stage of the parasite is the formation of a cyst within infected cells, allowing the parasite to escape the host’s immune response. In this study, a new bradyzoite cyst matrix antigen (MAG) was identified through a hybridoma library screen. This cyst matrix antigen, matrix antigen 2 (MAG2), contains 14 tandem repeats consisting of acidic, basic, and proline residues. Immunoblotting revealed that MAG2 migrates at a level higher than its predicted molecular weight, and computational analysis showed that the structure of MAG2 is highly disordered. Cell fractionation studies indicated that MAG2 was associated with both insoluble and soluble cyst matrix material, suggesting that it interacts with the intracyst network (ICN). Examination of the kinetics of MAG2 within the cyst matrix using fluorescence recovery after photobleaching (FRAP) demonstrated that MAG2 does not readily diffuse within the cyst matrix. Kinetic studies of MAG1 demonstrated that this protein has different diffusion kinetics in tachyzoite and bradyzoite vacuoles and that its mobility is not altered in the absence of MAG2. In addition, deletion of MAG2 does not influence growth, cystogenesis, or cyst morphology.

**IMPORTANCE** This report expands on the list of characterized Toxoplasma gondii cyst matrix proteins. Using fluorescence recovery after photobleaching (FRAP), we have shown that matrix proteins within the cyst matrix are not mainly in a mobile state, providing further evidence of how proteins behave within the cyst matrix. Understanding the proteins expressed during the bradyzoite stage of the parasite reveals how the parasite functions during chronic infection.

## Abstract (precis)

This report expands on the list of characterized Toxoplasma gondii cyst matrix proteins. Using fluorescence recovery after photobleaching (FRAP), we have shown that matrix proteins within the cyst matrix are not mainly in a mobile state, providing further evidence of how proteins behave within the cyst matrix. Understanding the proteins expressed during the bradyzoite stage of the parasite reveals how the parasite functions during chronic infection.

## INTRODUCTION

Exposure to contaminated food containing bradyzoites, which represent the latent stage of Toxoplasma gondii, or to oocysts containing sporozoites, which represent the sexual stage of the parasite, results in the transmission of this parasite (1). In the case of pregnant mothers, first-time exposure may lead to complications within the unborn fetus (2). While the levels of seroprevalence of T. gondii differ from country to country (3), it is estimated that 30% to 40% of the human population is latently infected with this parasite. There is currently no cure for this infection in the latent stage, and it is believed that this organism can persist for life in its hosts. The persistence of the infection is due to the ability of the parasite to differentiate from tachyzoites, representing the quickly proliferating stage, into bradyzoites, which can remain dormant within cysts for many years. When the host’s immune system is weakened, the parasite can revert to the tachyzoite stage and reactivate, causing symptoms such as encephalitis in reactivated toxoplasmosis (4).

The cyst has been proposed to establish chronic infection within its host by protecting the parasite from host immune responses and to shield the parasite from harsh digestive environments during ingestion, enhancing transmissibility of the parasite (5, 6). Studying the protein components of the cyst has allowed researchers to begin to understand the biology of the cyst and its function for the parasite (7–10). Matrix antigen 1 (MAG1) has been previously described to localize to the cyst wall and cyst matrix (11), which contain dense osmiophilic material, vesicles of various sizes, tubular structures connecting bradyzoites, and filamentous material (12). Like dense-granule proteins (GRAs), MAG1 is not exclusively expressed during the bradyzoite stage (13). Other proteins such as CST1 (7, 14), BPK1 and MCP4 (15), various GRAs (16), and cyst wall proteins (CSTs) (9) have been shown to localize to the cyst matrix and/or the cyst wall. Deletion of GRA4, GRA6 (17), BPK1 (10), and CST1 (7) has previously been shown to result in diminished cyst numbers during *in vivo* mouse chronic infection. In addition, CST1 has been shown to be important for the robustness of cysts with respect to withstanding physical, mechanical stress, for the maintenance of bradyzoite gene expression, and for the formation of the cyst wall as observed with transmission electron microscopy (TEM). While CST1 is a crucial component of the cyst wall, parasites with CST1 deleted (ΔCST1 strains) are still able to form cysts, albeit smaller and more fragile ones (7).

To investigate the components of the residual cyst wall of ΔCST1 parasites, a hybridoma library created from cyst lysates of the type II ME49 strain was screened against the residual cyst wall of ΔCST1 parasites by immunofluorescence assay (IFA). A monoclonal antibody (MAb) that reacted mainly to the matrix of T. gondii cysts was isolated, and the antigen that it recognized was identified and named matrix antigen 2 (MAG2), based on its localization being primarily in the cyst matrix rather than in the cyst wall. To characterize MAG2, the locus harboring the gene encoding MAG2 was edited using CRISPR-Cas9 to knock out this gene and the phenotype of the resulting ΔMAG2 parasites was characterized. In addition, MAG2 was investigated for its diffusion kinetics within the cyst matrix using fluorescence recovery after photobleaching (FRAP), which demonstrated that MAG2 displays limited diffusion within the cyst matrix.

## RESULTS

### 20C3 MAb recognizes a bradyzoite matrix antigen.

**FIG 1:**
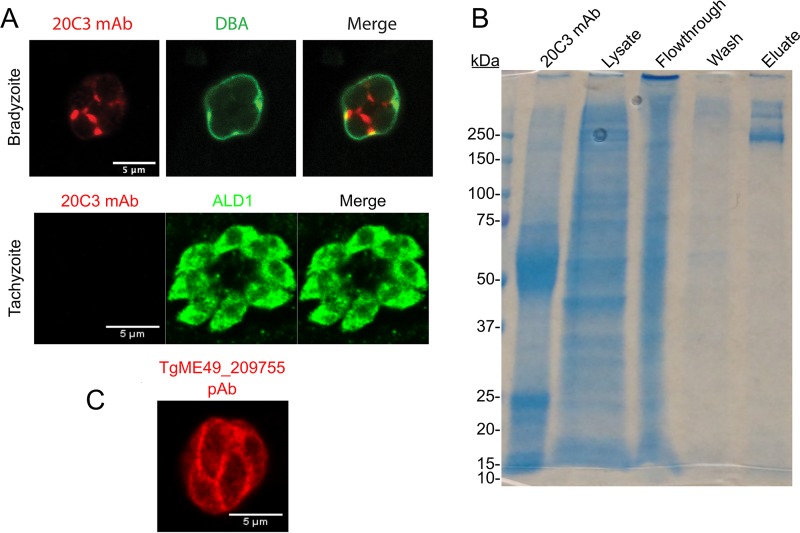
20C3 MAb recognizes a bradyzoite matrix antigen. (A) Pru Δ*ku80* Δ*hxgprt* (Pru) parasites were inoculated into HFF host cells and incubated under pH 8 conditions for 3 days for bradyzoite differentiation (top) or were maintained under normal cell culture conditions for tachyzoite growth (bottom) before fixation for IFA was performed. Parasites were stained with 20C3 monoclonal antibody (MAb) and fluorescein-labeled Dolichos biflorus agglutinin (DBA) to identify bradyzoite vacuoles or with aldolase 1 (ALD1) to visualize the parasite body. (B) Immunoprecipitation of *in vitro* bradyzoite lysates with the 20C3 MAb was analyzed on SDS-PAGE by staining with Coomassie blue. The bands within the eluate lane were sent to mass spectrometry for identification of the 20C3 MAb antigen.

To identify antigens of the residual cyst wall of ΔCST1 parasites, a library of monoclonal antibodies was generated using cyst wall lysates from ME49 parasites as the immunogen. This library was screened against ΔCST1 parasites by immunofluorescence assay (IFA), and monoclonal antibody (MAb) clone 20C3 was selected for further characterization as it demonstrated an interesting cyst matrix staining pattern with a slight cyst wall localization in bradyzoite vacuoles and no staining in tachyzoite vacuoles (Fig. 1A). The 20C3 MAb was subcloned and concentrated for immunoprecipitation. Pulldown of bradyzoite antigens from the Pru Δ*ku80* Δ*hxgprt* (Pru) strain using 20C3 MAb demonstrated three bands on SDS-PAGE (Fig. 1B; eluate lane). These bands were submitted for protein analysis by liquid chromatography-electrospray ionization-tandem mass spectrometry (LC-ESI-MS/MS), which revealed the major T. gondii protein to be a hypothetical protein, T. gondii ME49_209755 (TgME49_209755). This protein was also identified in a cyst wall proteomic preparation (9). To further assess whether TgME49_209755 is the antigen recognized by 20C3 MAb, a murine polyclonal antibody (PAb) was created by immunizing mice with peptides expressed from the coding sequence of TgME49_209755. IFA demonstrated that this polyclonal antibody also stained the matrix of bradyzoites (Fig. 1C). Time course IFA of stained parasite cultures demonstrated that no staining with 20C3 MAb was observable in tachyzoites at 6, 24, and 48 h postinfection or at 6 and 24 h after bradyzoite differentiation. Staining with 20C3 MAb in IFA (see Fig. S1 in the supplemental material) and immunoblotting (data not shown) was observed starting at 48 h postdifferentiation. Transcriptome sequencing (RNA-seq) data on ToxoDB.org did, however, demonstrate some mRNA expression for TgME49_209755 at 24 h postdifferentiation. As TgME49_209755 primarily displayed localization to the cyst matrix rather than to the cyst wall, this gene was named the matrix antigen 2 (MAG2) gene.

10.1128/mSphere.00100-20.1 FIG S1 20C3 MAb recognizes a bradyzoite matrix antigen starting at 48 h postdifferentiation. Pru Δ*ku80* Δ*hxgprt* (Pru) parasites were inoculated into HFF host cells and incubated under pH 7 (left) or pH 8 conditions for bradyzoite differentiation (right) for 6, 24, or 48 h. Parasites were stained with 20C3 MAb and an aldolase 1 (ALD1) antibody or an anti-Toxo antibody. Download FIG S1, PDF file, 0.2 MB. Copyright © 2020 Tu et al. 2020 Tu et al. This content is distributed under the terms of the Creative Commons Attribution 4.0 International license.

### Confirmation of MAG2 as the antigen recognized by the 20C3 MAb.

**FIG 2:**
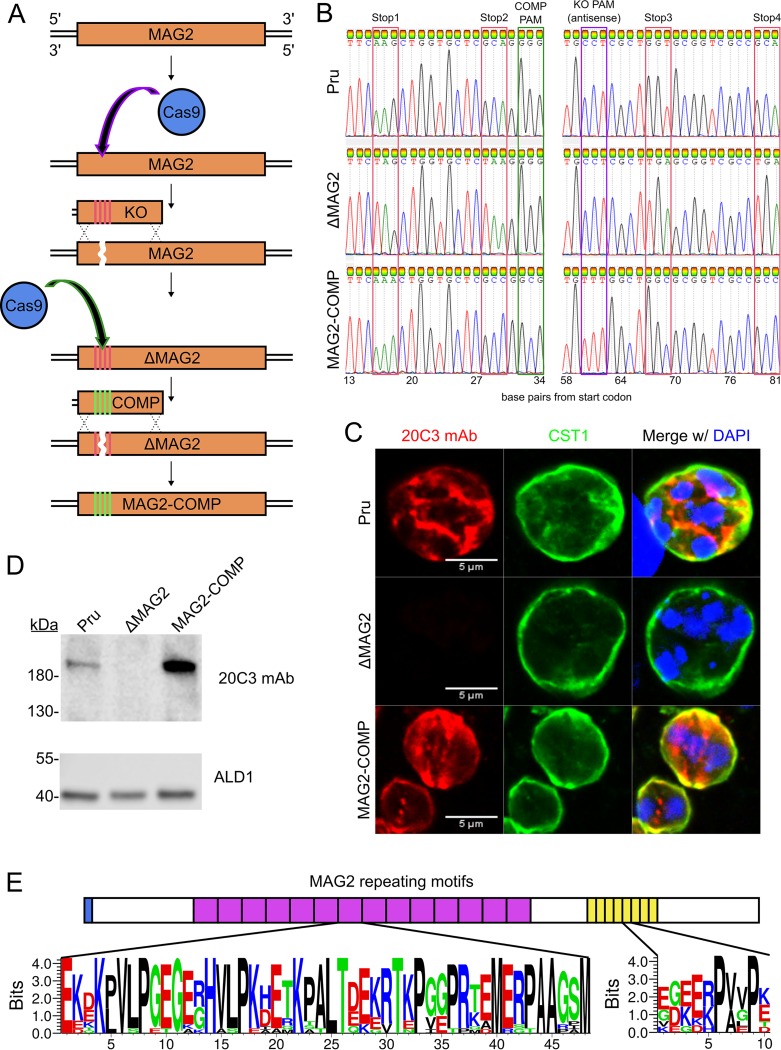
20C3 MAb recognizes MAG2, a disordered 218-kDa protein with tandem repeats. (A) Schematic showing the endogenous editing of the TgME49_209755 (MAG2) locus. Red bars indicate regions with inserted stop codons, while green bars indicate regions with synonymous mutations. KO, knockout; COMP, complementation. (B) Results of sequencing of the MAG2 N terminus for the Pru, ΔMAG2, and MAG2-COMP strains. The red boxes depict the DNA sequences within the MAG2 locus where stop codons were introduced to generate ΔMAG2. These stop codons were subsequently removed to create the MAG2-COMP strain. Protospacer-adjacent-motif (PAM) sites for the knockout and COMP sgRNAs are highlighted in purple and green boxes, respectively. These PAM sites were intentionally mutated in the MAG2-COMP strain. (C) IFA results showing *in vitro* Pru (top), ΔMAG2 (middle), and MAG2-COMP (bottom) bradyzoites differentiated for 3 days and stained with 20C3 MAb and anti-CST1. (D) Lysates from Pru, ΔMAG2, and MAG2-COMP strains were probed on immunoblots by 20C3 MAb (upper panel). The lower panel (ALD1) shows the loading control. (E) Seq2Logo representation of the repeating domains of MAG2. The blue box represents the signal peptide, while an individual purple or yellow box represents a single repeat of 48 or 10 amino acids, respectively. The Seq2Logo represents the frequency of the observed amino acid within a single repeat. Large symbols indicate more frequently observed amino acids. The Seq2Logo was constructed by aligning the 48 amino acid repeats (purple) and the 10 amino acid repeats (yellow) and generating a nonweighted Shannon logo without clustering. Seq2Logo default amino acid color coding is used (DE residues are red, NQSGTY residues are green, RKH residues are blue, and the remaining residues are black).

To validate that the protein recognized by 20C3 MAb is MAG2 (TgME49_209755), this protein was knocked out by inserting premature stop codons into the N terminus of the gene to create MAG2 knockout (Pru Δ*mag2*) parasites. Subsequently, the complement strain (MAG2-COMP) of the ΔMAG2 parasite was generated by removing the premature stop codons and replacing them with synonymous mutations by CRISPR-Cas9 (Fig. 2A). Generation of the ΔMAG2 and complementation strains was validated by Sanger sequencing (Fig. 2B). No staining with 20C3 MAb was observed by IFA in the ΔMAG2 parasite (Fig. 2C, middle panel) until the gene was complemented back (Fig. 2C, bottom panel). Additionally, immunoblotting of the Pru and MAG2-COMP strains with 20C3 MAb revealed a band at 218 kDa which disappeared in the ΔMAG2 strain (Fig. 2D), validating that 20C3 MAb recognizes MAG2 as its antigen. The higher level of expression of MAG2 within the MAG2-COMP strain than within the Pru strain may have been due to the synonymous mutations introduced into MAG2 in the MAG2-COMP strain, which have had better codon optimization than wild-type MAG2. Unpublished data on tRNA abundance and protein levels as determined in our laboratory indicate that codon usage for amino acid residues can alter protein expression levels (Silmon de Monerri, unpublished data).

### The repeating region in MAG2 results in its higher-molecular-weight shift.

The 218-kDa band of MAG2 revealed by immunoblotting (Fig. 2D) and SDS-PAGE (Fig. 1B) represents a molecular weight that is ∼78 kDa higher than the predicted molecular weight of MAG2. Mass spectrometry analysis of the 20C3 MAb immunoprecipitation data revealed no potential posttranslational modifications of MAG2 to explain this shift from its predicted molecular weight. The MAG2 amino acid sequence has 14 long tandem repeats of 48 amino acids (aa) (Fig. 2E) that are rich in acidic, basic, and proline residues, and the repeat region is predicted to represent a disordered domain (Fig. 2D) (18). Deletion of the MAG2 repeat region eliminated aberrant migration (Fig. S2), as demonstrated by migration of a truncated version of MAG2 (trMAG2-BirA*-3×HA; lacking repeats 2 to 12 and containing C-terminal BirA* [19] and hemagglutinin [HA] tags) at its predicted molecular weight (140 kDa). This supports the hypothesis that the aberrant migration of MAG2 is likely a consequence of the presence of the predicted disordered MAG2 repeat region (Fig. 2D and E).

10.1128/mSphere.00100-20.2 FIG S2 Tandem repeating regions of MAG2 migrate at levels higher than its predicted molecular weight. (A) Schematic showing the resulting truncation of MAG2 after tagging with BirA* and 3×HA. (B) DNA electrophoresis on a 1% agarose gel of PCR products amplified from the 5′UTR of MAG2 and coding sequence using MAG2-specific primers on Pru genomic DNA (Pru gDNA) or the plasmid containing truncated MAG2 tagged with BirA* and 3×HA (p-trMAG2-BirA*-3×HA). (C) Lysates from Pru and truncated MAG2 (trMAG2-BirA*-3×HA) parasites were probed by immunoblotting with 20C3 MAb and anti-HA antibody. Transfection of the trMAG2-BirA*-3×HA plasmid into the Pru strain yielded parasites that exogenously expressed the truncated version of MAG2 in addition to expression of the wild-type MAG2. (D) Protein disorder prediction of MAG2 using PrDOS. The coding sequence of wild-type MAG2 contains 14 tandem long repeats of 48 amino acids in the middle of the gene. Truncation of the MAG2 coding sequence (missing repeats 2 to 12 detected by sequencing) was found to have occurred in the trMAG2-BirA*-3×HA construct and probably occurred during culture of the transformed E. coli strain. The trMAG2-BirA*-3×HA protein migrated at 140 kDa. Full-length unlabeled MAG2 has a migration mass of 218 kDa, and a full-length MAG2 tagged with BirA* (36 kDa) and 3×HA (3 kDa) should, therefore, have a migration mass of 257 kDa. Based on this, the missing 11 repeats in the truncated MAG2 have a migration mass of 117 kDa (i.e., 257 kDa − 140 kDa), which represents a mass of ∼10.6 kDa per repeat. This migration mass is approximately double the ∼5.2 kDa per repeat that was predicted on the basis of their amino acid composition. This aberrant migration of the 14-repeat region (an additional 76 kDa) can explain the observed migration of full-length MAG2 at ∼218 kDa. Download FIG S2, PDF file, 0.3 MB. Copyright © 2020 Tu et al. 2020 Tu et al. This content is distributed under the terms of the Creative Commons Attribution 4.0 International license.

### MAG2 does not readily diffuse in the cyst matrix.

**FIG 3:**
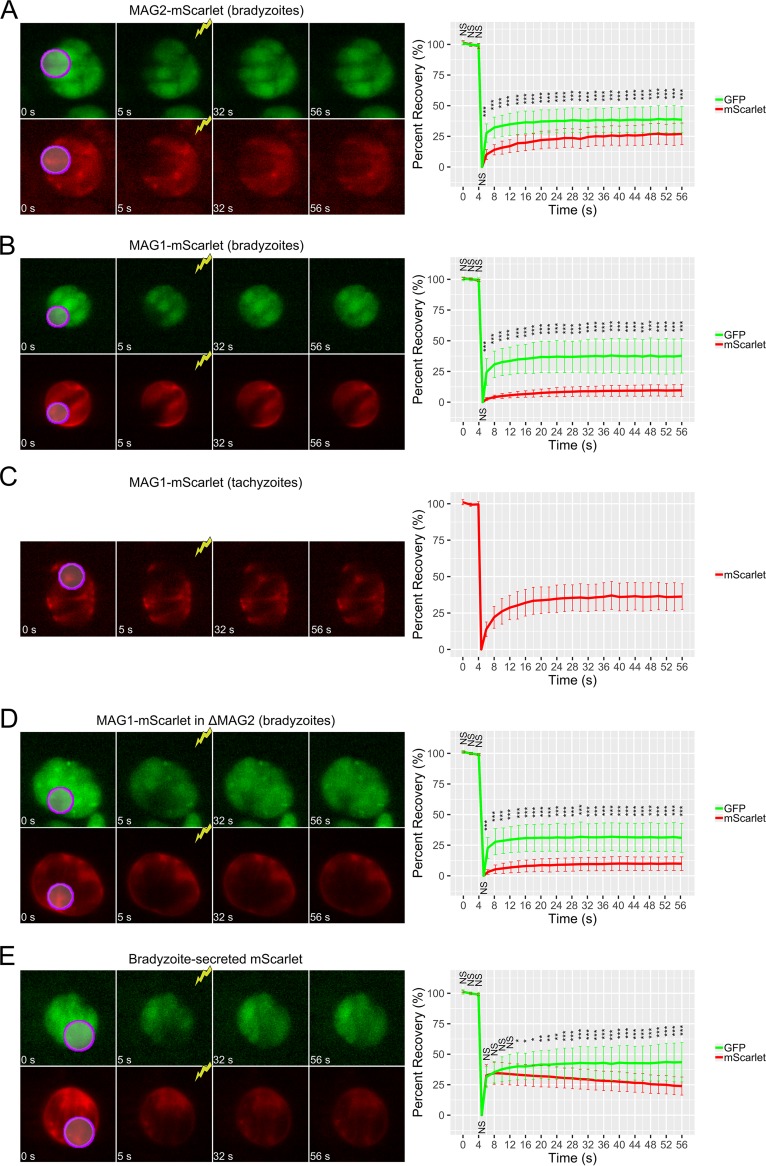
Matrix proteins do not readily diffuse throughout the cyst. (Left panels) FRAP time-lapse images of *in vitro* (A) MAG2-mScarlet bradyzoites, (B) MAG1-mScarlet bradyzoites, (C) MAG1-mScarlet tachyzoites, (D) MAG1-mScarlet bradyzoites with MAG2 deletion, and (E) bradyzoites expressing mScarlet under the control of the CST1 5′UTR. All bradyzoites were induced for 4 days. The bleached areas are shown in purple. (Right panels) Relative percentages of recovery of the bleached fluorophore. The percentage of recovery of mScarlet was compared to that of GFP at each time point (NS, *P* > 0.05; *, *P* < 0.05; **, *P* < 0.01; ***, *P* < 0.001). A total of 21 cysts from each strain were analyzed.

Studies on the diffusion kinetics of T. gondii cysts have been limited to the use of small molecules conjugated to fluorophores to observe the permeability of the cyst wall and the diffusion of molecules within the cyst (12). To study parasite protein diffusion within the cyst, endogenous MAG2 was tagged C-terminally with mScarlet (MAG2-mScarlet). MAG2-mScarlet parasites were differentiated to bradyzoites, and the cyst was photobleached to see the recovery of endogenous MAG2 together with the use of a LDH2 driven cytosolic green fluorescent protein (GFP) (20) as a soluble mobile protein control. Fluorescence recovery after photobleaching (FRAP) analysis revealed that MAG2-mScarlet did not readily recover into the bleached area and had an immobile fraction (the percentage of the protein that does not diffuse) of 73.28% ± 9.67% with a half-life of equilibrium of 4.08 s ± 1.79 s (Fig. 3A) (Table 1; see also Movie S1 in the supplemental material). On the other hand, the cytosolic GFP had an immobile fraction of 58.86% ± 11.52% with a half-life of equilibrium of 1.12 s ± 0.73 s, which represent statistically significant differences from the FRAP values determined for MAG2-mScarlet (Table 1). To investigate if the limited movement of MAG2 within the cyst matrix was restricted to MAG2 alone, MAG1 was also tagged endogenously on its C terminus with mScarlet (MAG1-mScarlet) for FRAP analysis. Interestingly, MAG1-mScarlet bradyzoites also displayed limited recovery of MAG1 after photobleaching of the cyst (Fig. 3B) (Table 1; see also Movie S2); in contrast, when the tachyzoite vacuole was photobleached, MAG1-mScarlet was more mobile (Fig. 3C) (Table 1; see also Movie S3). To test if the limited mobility of MAG1 in the bradyzoite stage is dependent on the presence of MAG2 (which is expressed in bradyzoite vacuoles), ΔMAG2 parasites had their endogenous MAG1 gene C-terminally tagged with mScarlet. FRAP analysis of MAG1-mScarlet within the ΔMAG2 background still demonstrated limited recovery of MAG1-mScarlet, suggesting that MAG1’s immobility in the bradyzoite stage is independent of MAG2 (Fig. 3D) (Table 1; see also Movie S4). To address if the limited recovery of MAG1 and MAG2 within bradyzoite cysts could have been due to mScarlet binding to immobile structures within the cyst, a bradyzoite-specific secreted form of mScarlet was constructed by ligating mScarlet to the promoter and signal peptide of CST1 (Cyst-matrix-mScarlet) and expressed within Pru. Cyst-matrix-mScarlet parasites demonstrated increased rates of recovery of mScarlet after photobleaching that were comparable to the rates seen with cytosolic GFP (Fig. 3E) (Table 1; see also Movie S5). However, as mScarlet is not as stable as the superfolded GFP, the mScarlet intensity decreased over time after each imaging step. These FRAP experiments demonstrated that whereas the matrices of the tachyzoite and bradyzoite vacuoles were permissible with respect to protein diffusion, the movement of MAG1 and MAG2 was more restricted in the bradyzoite vacuole than was found to be the case with the soluble bradyzoite-secreted mScarlet fluorescent protein.

**TABLE 1:** Analysis of FRAP parameters^*a*^

Protein of interest and FRAP parameter	mScarlet values	Cytosolic GFP values	*P* value
MAG2 (pH 8)			
Immobile fraction (%)	73.28 ± 9.67	58.86 ± 11.52	<0.0001
Time of equilibrium (s)	4.08 ± 1.79	1.12 ± 0.73	<0.000001

MAG1 (pH 8)			
Immobile fraction (%)	89.92 ± 5.06	61.85 ± 15.34	<0.0000001
Time of equilibrium (s)	4.87 ± 2.20	1.47 ± 0.80	<0.000001

MAG1 (pH 7)			
Immobile fraction (%)	61.72 ± 8.29		
Time of equilibrium (s)	3.79 ± 2.36		

MAG1 (pH 8) in MAG2-KO			
Immobile fraction (%)	89.60 ± 5.92	66.77 ± 11.80	<0.00000001
Time of equilibrium (s)	3.95 ± 1.96	0.97 ± 0.050	<0.000001

Secreted mScarlet (pH 8)			
Immobile fraction (%)	63.40 ± 7.91	54.66 ± 15.71	0.030
Time of equilibrium (s)	0.88 ± 0.09	1.16 ± 0.78	0.115

a FRAP parameter data include mean percentages of immobile fractions and times of equilibrium and their standard deviations for mScarlet and cytosolic GFP across all bleaching experiments. Cytosolic GFP (LDH2-GFP) is not expressed at pH 7. The Welch two-sample *t* test was used to compare the mean immobile fractions or the times of equilibrium between the mScarlet and GFP fluorophores (*n* = 21 for each experiment).

10.1128/mSphere.00100-20.6 MOVIE S1 Movie of a representative FRAP experiment performed on *in vitro* MAG2-mScarlet bradyzoites. The video is played at 1 frame per second (fps), and the time is indicated in seconds. Download Movie S1, AVI file, 4.5 MB. Copyright © 2020 Tu et al. 2020 Tu et al. This content is distributed under the terms of the Creative Commons Attribution 4.0 International license.

10.1128/mSphere.00100-20.7 MOVIE S2 Movie of a representative FRAP experiment performed on *in vitro* MAG1-mScarlet bradyzoites. The video is played at 1 frame per second (fps), and the time is indicated in seconds. Download Movie S2, AVI file, 4.5 MB. Copyright © 2020 Tu et al. 2020 Tu et al. This content is distributed under the terms of the Creative Commons Attribution 4.0 International license.

10.1128/mSphere.00100-20.8 MOVIE S3 Movie of a representative FRAP experiment performed on *in vitro* MAG1-mScarlet tachyzoites. The video is played at 1 frame per second (fps), and the time is indicated in seconds. Download Movie S3, AVI file, 1.5 MB. Copyright © 2020 Tu et al. 2020 Tu et al. This content is distributed under the terms of the Creative Commons Attribution 4.0 International license.

10.1128/mSphere.00100-20.9 MOVIE S4 Movie of a representative FRAP experiment performed on *in vitro* MAG1-mScarlet bradyzoites with MAG2 deleted. The video is played at 1 frame per second (fps), and the time is indicated in seconds. Download Movie S4, AVI file, 4.5 MB. Copyright © 2020 Tu et al. 2020 Tu et al. This content is distributed under the terms of the Creative Commons Attribution 4.0 International license.

10.1128/mSphere.00100-20.10 MOVIE S5 Movie of a representative FRAP experiment performed on *in vitro* bradyzoites expressing mScarlet under the control of the CST1 5′UTR and signal peptide. The video is played at 1 frame per second (fps), and the time is indicated in seconds. Download Movie S5, AVI file, 4.5 MB. Copyright © 2020 Tu et al. 2020 Tu et al. This content is distributed under the terms of the Creative Commons Attribution 4.0 International license.

To determine if MAG2 is a soluble or insoluble (i.e., membrane-associated) protein, cell fractionation of infected human foreskin fibroblasts (HFFs) was performed using methods employed for the characterization of GRA1 (21) and of GRA2 and GRA5 (22) (Fig. S3). After lysis performed with a 27.5-gauge needle, intact parasites were separated from cyst material by low-speed centrifugation. While MAG2 was detected in the low-speed supernatant (LSS) containing cyst material, the low-speed pellet (LSP) also contained MAG2. Detection of MAG2 in this pellet may have been due to the presence of intact cysts that were not lysed or to bradyzoite expression of unsecreted MAG2. The LSS was subjected to high-speed centrifugation to separate soluble from insoluble cyst material before the insoluble cyst material was treated with buffers to disrupt electrostatic interactions. MAG2 was detected as a soluble form in the untreated high-speed supernatant (HSS) and as an insoluble form in phosphate-buffered saline (PBS) and in the high-speed pellet (HSP) after treatment with 1 M NaCl. The presence of MAG2 in the HSS and HSP after centrifugation of the LSS is similar to how GRA1 behaves in the intravacuolar network (IVN) of tachyzoites (21). This suggests that MAG2 is associated with the intracyst network (ICN) in bradyzoite vacuoles. The ICN in bradyzoite vacuoles is similar to the IVN seen in tachyzoite vacuoles (12). Unlike GRA5 (an integral membrane protein [22]), MAG2 (which has no predicted membrane association region) was able to be disrupted from the HSP with salt buffers such as PBS and 1 M NaCl, suggesting that MAG2 associates with insoluble cyst material through electrostatic interactions. MAG1 also displayed a fractionation pattern similar to that seen with MAG2. As a control for parasite contamination, SAG1 surface protein was also assessed and shown to appear mainly in the LSP. Taken together, the data suggest that MAG2 and MAG1 have electrostatic associations with insoluble cyst material but that they also retain soluble forms within the matrix. The data revealing an association with the insoluble fraction are consistent with the mobility data seen in FRAP analysis of MAG2.

10.1128/mSphere.00100-20.3 FIG S3 Membrane fractionation of infected HFFs. HFFs infected with Pru bradyzoites (pH 8 induction for 3 days) or tachyzoites (pH 7) were lysed and centrifuged using low speeds to separate host cell debris and cyst/parasitophorous material from intact parasites. The supernatant obtained at the low speeds was further fractionated by high-speed (100,000 × *g*) centrifugation, and the resulting pellet obtained at high speed was treated with PBS or 1 M NaCl salts before being centrifuged again at high speeds. Each fraction was probed with 20C3 MAb or with anti-MAG1 or anti-SAG1 antibody (parasite control) on an immunoblot. LSS, low-speed supernatant; LSP, low-speed pellet; HSS, high-speed supernatant; HSP, high-speed pellet. Download FIG S3, PDF file, 0.1 MB. Copyright © 2020 Tu et al. 2020 Tu et al. This content is distributed under the terms of the Creative Commons Attribution 4.0 International license.

### Characterization of Δ*MAG2* Toxoplasma gondii.

Growth of Pru, ΔMAG2, and MAG2-COMP parasites was analyzed by plaque assay (Fig. S4A and B). No differences in plaque size were observed among these three strains, suggesting that MAG2 plays no role in parasite replication. The parental Pru and the recreated ΔMAG2 parasite strains were injected into C57BL/6 mice (*n* = 10 per group). The fitness scores for MAG2 (0.59 for TgME49_209755A and 1.12 for TgME49_209755B) reported at ToxoDB.org are consistent with this gene being nonessential (57). Analysis of mouse survival during acute infection (14 days) revealed that this new ΔMAG2 strain did not kill significantly more mice than the Pru parental strain (*P* = 0.46) (Fig. S4C). These data are consistent with the fact that there is no protein expression of MAG2 (as detected by IFA) in tachyzoites (Fig. S1), despite some evidence represented by RNA-seq data available at ToxoDB.org of transcripts being present in tachyzoites. The presence of transcripts without detectable protein expression in tachyzoites may reflect the presence of a gene that is ready for translation during bradyzoite differentiation or a subset of parasites undergoing transition from tachyzoites to bradyzoites in the RNA-seq samples or a very short protein half-life for MAG2 protein in tachyzoites.

10.1128/mSphere.00100-20.4 FIG S4 Characterization of ΔMAG2 Toxoplasma gondii. (A) Representative plaque assays of Pru, ΔMAG2, and MAG2-COMP parasites. (B) Quantification of plaque sizes from each strain. (C) Survival curve of mice challenged with Pru and recreated ΔMAG2 parasites (*n* = 10 for each strain; *P* = 0.46). Download FIG S4, PDF file, 0.2 MB. Copyright © 2020 Tu et al. 2020 Tu et al. This content is distributed under the terms of the Creative Commons Attribution 4.0 International license.

**FIG 4:**
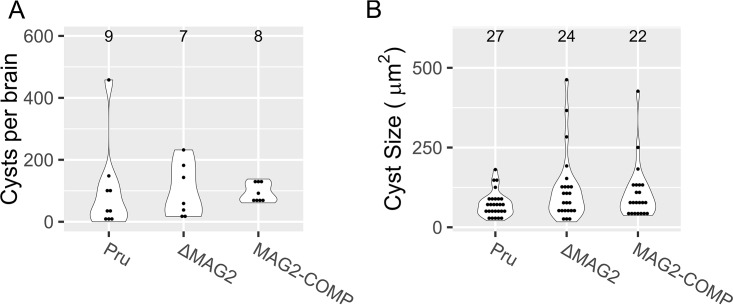
Characterization of the ΔMAG2 strain reveals no defects in cyst burden or cyst size. (A) Brain cyst counts of mice infected with Pru, ΔMAG2, and MAG2-COMP strains at 30 days postinfection. The number of brains counted is listed above each violin plot. (B) Size analysis of *in vivo* cysts obtained from Pru, ΔMAG2, and MAG2-COMP strains. The number of cysts imaged and analyzed is listed above each violin plot. No significant differences in cyst numbers or cyst sizes were observed in the comparisons between these three strains (*P* > 0.05).

To observe if MAG2 affects cystogenesis, Pru, ΔMAG2, and MAG2-COMP strains were used to infect C57BL/6 mice and brains from these mice were harvested after 30 days. Cyst counts of infected brain homogenates demonstrated that MAG2 did not affect the ability of T. gondii to form cysts *in vivo* and did not affect the number of cysts seen in the brains of mice following infection; e.g., there were no significant differences seen between the Pru parental, ΔMAG2, and MAG2-COMP strains (Fig. 4A). In addition, the sizes of cysts retrieved from these strains were not significantly different (Fig. 4B).

### Cyst morphology of the MAG2 strains.

**FIG 5:**
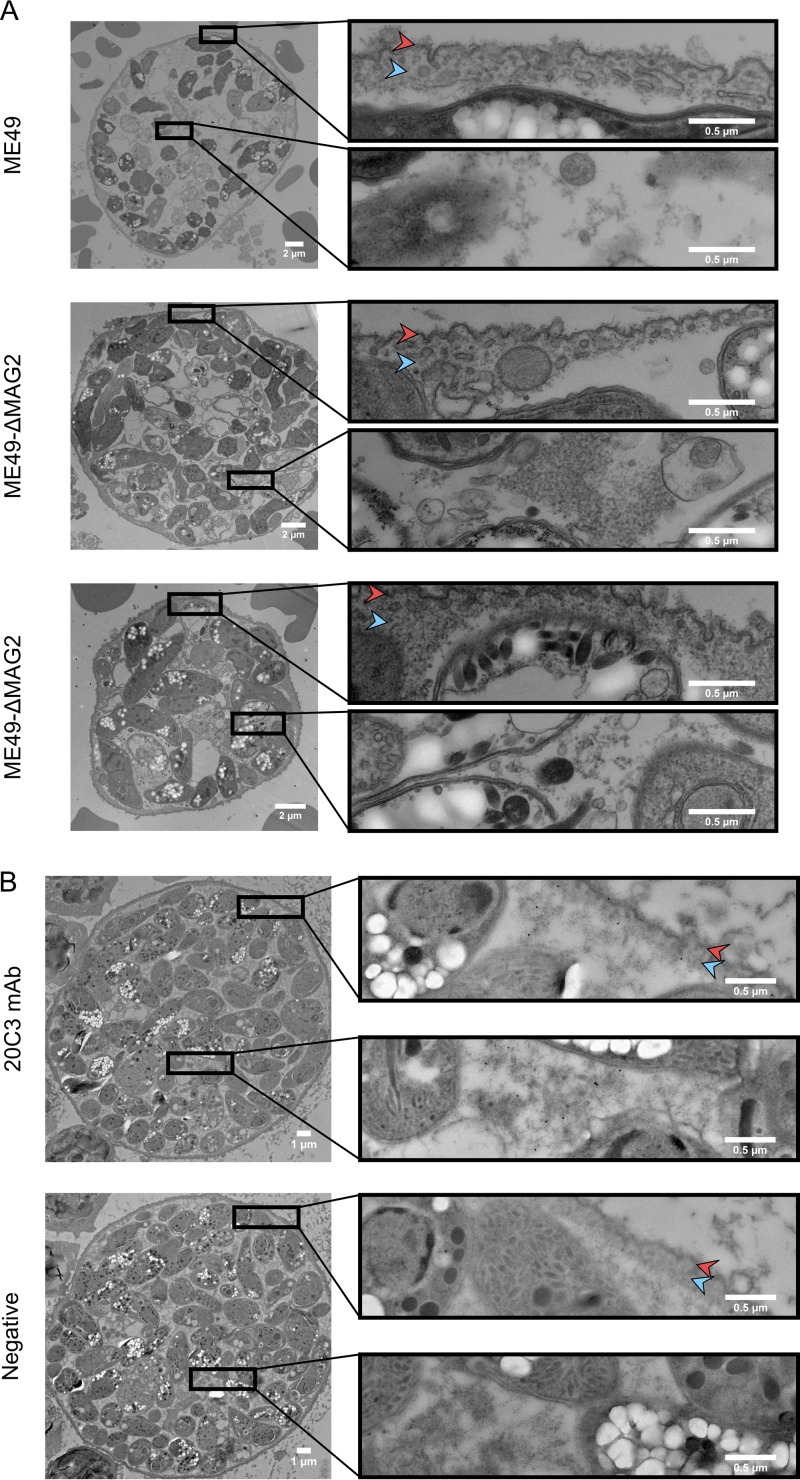
MAG2 does not affect cyst morphology. (A) Electron micrographs of *in vivo* cysts from the parental ME49 Δ*hxgprt* Δ*ku80* (ME49) strain (top) and ME49 Δ*hxgprt* Δ*ku80* Δ*mag2* (ME49-ΔMAG2) strains (middle and bottom). (B) Immunogold electron micrograph of an *in vivo* ME49 cyst stained with 20C3 MAb primary antibody (top) or left unstained (bottom). Red arrowheads point to the cyst membrane, while blue arrowheads point to the cyst wall.

In order to generate enough cysts for transmission electron microscopy, MAG2 was knocked out in ME49 Δ*hxgprt* Δ*ku80* parasites to create the ME49 Δ*hxgprt* Δ*ku80* Δ*mag2* strain. These strains were injected into BALB/c^Δdm1^ mice intraperitoneally, and cysts were harvested after 30 days. Morphological transmission electron microscopy analysis of these *in vivo* cysts revealed no morphological differences within the cyst and or the cyst wall between these strains (Fig. 5A). In addition, the role of MAG2 in the formation of the cyst wall of the Pru strain was analyzed by preparing *in vitro* cysts of the Pru, ΔMAG2, and MAG2-COMP strains; *in vitro* cysts generated from these strains also revealed no distinguishing differences in the cyst morphologies of the Pru and ΔMAG2 strains (Fig. S5). There were, however, a few focal areas with subtle changes seen in the cyst wall and cyst matrix in the MAG2-COMP strain (Fig. S5) which may have been due to the overexpression of MAG2 in this strain (Fig. 2D).

10.1128/mSphere.00100-20.5 FIG S5 The morphology of ΔMAG2 *in vitro* cysts is unaltered. Electron micrographs of *in vitro* cysts from the parental Pru (top), ΔMAG2 (middle), and MAG2-COMP (bottom) strains are presented. Red arrows point to the cyst membrane, while the blue arrows point to the cyst wall. Download FIG S5, PDF file, 0.6 MB. Copyright © 2020 Tu et al. 2020 Tu et al. This content is distributed under the terms of the Creative Commons Attribution 4.0 International license.

To examine localization of MAG2 at the ultrastructural level, ME49 *in vivo* cysts were prepared and stained for immunogold electron microscopy. Localization of MAG2 could be seen within the cyst matrix and in proximity to the cyst wall within the ME49 strain (Fig. 5B).

## DISCUSSION

Tour de force approaches such as screenings using libraries of monoclonal antibodies (7, 23) and cDNA (11, 24) have led to the identification and characterization of crucial bradyzoite antigens such as BAG1, MAG1, and CST1. While CST1 has been found to be important for maintaining cyst wall integrity and establishment of chronic infection, ΔCST1 parasites still retain the ability to create cysts composed of a thin cyst wall. The remaining proteins that form this residual cyst wall were screened for using a monoclonal antibody library generated from wild-type ME49 cysts rather than from ΔCST1 cysts, which could not be harvested in large enough quantities to produce a monoclonal library. This approach yielded the 20C3 MAb, which reacts to the matrix of ΔCST1 cysts (image not shown). When the Pru strain was probed with this antibody by the use of immunofluorescence, matrix localization was also observed. Subsequently, the cyst matrix antigen to the 20C3 MAb was found to be the hypothetical protein TgME49_209755 and was renamed MAG2. In a subsequent study that examined the cyst proteome, we were also able to identify MAG2, confirming its presence in cysts (9).

To characterize MAG2, the endogenous locus was edited to knock out and to complement the gene. A limitation of this deletion strategy is that short N-terminal peptides from MAG2 may still be expressed in the ΔMAG2 strain and may interfere with phenotypic assays if these short peptides retain the functions of MAG2. Pru, ΔMAG2, and MAG2-COMP strains showed no significant differences in parasite replication, virulence, cystogenesis, or cyst morphology.

Because MAG2 does not exhibit much similarity to any other known protein, it remains difficult to identify the function of this protein. However, computational predictions based on the amino acid composition of MAG2 can provide insight on how this protein behaves. Signal peptide prediction (25) showed that there may be cleavage between aa 22 and 23 at the N terminus of MAG2, allowing MAG2 to be secreted into the matrix. In addition, primary sequence analysis shows that the amino acid composition of MAG2 consists of 39.8% hydrophobic residues, 20% acidic residues (16% Glu and 4% Asp), and 18% basic residues (7% Arg and 11% Lys). The 48 amino acid repeating sequences of MAG2 are 35% hydrophobic, 19% acidic (15% Glu and 4% Asp), and 21% basic (8% Arg and 13% Lys) and are interspersed with prolines (26). The high level of glycine content (8.4%) and high hydrophilicity index value classify MAG2 as a hydrophilin, which suggests that MAG2 may have a role in desiccation resistance (27). Furthermore, the middle region of MAG2 is predicted to be intrinsically disordered (see Fig. S2D in the supplemental material) (18), which also suggests a role for MAG2 in desiccation tolerance (28). The intrinsically disordered region on MAG2 also explains its aberrant electrophoretic mobility in SDS-PAGE (Fig. 1B), as intrinsically disordered proteins often show a molecular weight 1.2 to 1.8 times higher than their predicted weight in SDS-PAGE (29). Protein folding prediction based on the C terminus of MAG2 revealed alpha helices and coiled coils (30, 31), which suggests that MAG2 may have membrane associations (32). Finally, the central repeating domains of MAG2 share some homology with the repeating units of Futsch, a *Drosophila* microtubule-associated protein (33), which suggests that MAG2 may be interacting with filamentous structures within the matrix (12, 34).

The expression pattern of MAG2 is similar to that of bradyzoite markers such as LDH2, whereby its expression is upregulated in the bradyzoite stage (Fig. S1). Not surprisingly, MAG2 is also controlled by proteins regulating the developmental stages of the parasite that are called Apetala2 (AP2) transcription factors. Like other bradyzoite markers, MAG2 is upregulated when AP2IX-4 and AP2IX-9, bradyzoite gene repressors, are knocked out (35, 36). In addition, deletion of the bradyzoite activator AP2XI-4 downregulates MAG2 (37). Interestingly, other developmental regulators such as AP2IV-4 and AP2IV-3 do not alter MAG2 expression (38, 39). In addition to its designation as TgME49_209755, MAG2 was previously identified as TgME49_009750 and TgME49_009760, which have been combined into one open reading frame representing the same gene locus.

Transcriptomic analysis that inferred the presence of a gene regulatory network in the life cycle of T. gondii found MAG2 to be an externally regulated protein modulated by environmental cues (40). Other proteins within this group include NTPase II (41), which is predicted to supply energy to processes that occur in the vacuolar space. In addition, the gene regulatory network analysis assigned the MAG2 gene to the community of genes upregulated in the bradyzoite stage (40). Other genes included in this community include those encoding ENO1 (42), LDH2 (43), ANK1 (44), P-type ATPase (45), MCP3 (46), BPK1 (10), MAG1 (11), CST4 (9), and CST10 (47). The grouping of MAG2 with a community that participates in carbohydrate metabolic processes and cell redox homeostasis is intriguing and suggests that MAG2 might have a role in these processes (40).

A previous study looking at the permeability of the cyst wall showed that small molecules (less than 1 kDa in size) can easily diffuse through the cyst wall and then throughout the cyst but that molecules greater than 10 kDa in size cannot (12). We found that mScarlet (26 kDa) displayed rapid diffusion into the bleached area of the cyst, suggesting that mobility within the cyst matrix can occur for larger molecules (Fig. 3E). Tagged MAG1 and MAG2 mScarlet fusion proteins, however, are not readily recovered in the bleached regions, suggesting that they may be bound to immobile structures such as the filamentous or tubular structures of the intracyst network seen within the cyst matrix. Lemgruber et al. (12) reported that these electron dense, filamentous structures are observed only in the matrix of the cyst and are not present within the tachyzoite parasitophorous vacuole. This observation supports the idea of an association of MAG1 with these filamentous structures since the diffusion rate of MAG1-mScarlet is limited in the bradyzoite stage but not in the tachyzoite stage. MAG1 and MAG2 may associate with the intracyst network in a manner similar to how various GRAs associate with the intravacuolar network of the tachyzoite stage (48–50). The results of the fractionation experiment show that both MAG2 and MAG1 have soluble and insoluble forms (Fig. S3). However, the ratio of these two forms within the cyst cannot be reliably inferred from the fractionation data. Instead, based on the FRAP experiments, the major proportions of these two proteins within the cyst were present in the immobile fraction and had diffusion rates that were higher than those seen with GFP (Table 1). This suggests that they are mainly associated with insoluble cyst material, even though they have some soluble forms, consistent with the FRAP observations of limited mobility of MAG1 and MAG2 in the bradyzoite matrix. Interestingly, 61.72% of MAG1 was in the immobile fraction under pH 7 conditions, compared to 89.92% under pH 8 conditions. This fraction percentage of MAG1 under pH 7 conditions was similar to the immobile fraction of soluble GFP; however, under tachyzoite conditions, it takes MAG1 almost 3 times as long to diffuse based on its observed time of equilibrium.

In this study, a new cyst matrix protein was characterized and its kinetics within the cyst matrix was determined. While the function of MAG2 remains unknown, subsequent studies on MAG2 may start to characterize its function within the cyst. The tools developed in this study such as the 20C3 MAb and the fluorescently tagged strains may be useful in future characterizations of MAG2 and other cyst matrix proteins.

## MATERIALS AND METHODS

### Toxoplasma gondii cell culture and differentiation.

Host cells consisting of human foreskin fibroblasts (HFF) were cultured at pH 7 in Dulbecco’s modified Eagle’s medium (DMEM) containing 10% EquaFETAL bovine serum (Atlas Biologicals) and 5% penicillin-streptomycin (Life Technologies) at 5% CO_2_ and 37°C. Confluent HFF cells were inoculated with parasite strains for passaging using standard techniques (51). For differentiation into bradyzoites, parasites were inoculated into HFF cells and incubated for 2 h before replacement of the culture media with pH 8 DMEM containing 50 mM HEPES (Sigma-Aldrich), 1% penicillin-streptomycin, and 1% FBS and lacking sodium bicarbonate. Induced cultures were incubated at 37°C in the absence of CO_2_ for 3 to 7 days.

### Hybridoma generation and screening.

The bradyzoite antigen hybridoma library was generated as previously described (7). Briefly, BALB/c^dm1^ mice (52) were infected with ME49 parasites; after 4 weeks, brain cysts from infected mice were isolated by Percoll gradient ultracentrifugation (53). Isolated cysts were fractured by freeze-thaw cycles and emulsified with Freund’s complete adjuvant before subcutaneous injection into BALB/c mice. After 2 months, spleens from immunized mice were fused with myeloma cells to generate a hybridoma library (54). Using immunofluorescence assays (IFA), hybridoma supernatant was screened for reactivity against cysts from ΔCST1 parasites (7). Positive hybridoma clones were subcloned on agar plates and cultured in a CELLine Bioreactor (Wheaton) to increase antibody production.

### Immunoprecipitation (IP) of the 20C3 MAb antigen.

HFF cells were infected with Pru Δ*ku80* Δ*hxgprt* parasites at a multiplicity of infection (MOI) of 1. Parasites were differentiated to bradyzoites and lysed in radioimmunoprecipitation assay (RIPA) buffer (50 mM Tris [pH 7.5], 150 mM NaCl, 0.1% SDS, 0.5% sodium deoxycholate, 1% NP-40). Bradyzoite lysates were precleared by incubation with protein G agarose beads for 30 min at 4°C to remove nonspecifically bound proteins. Then, precleared samples were incubated with protein G agarose beads covalently cross-linked with clone 20C3 monoclonal antibody (MAb) by the use of bis(sulfosuccinimidyl)suberate (BS^3^; Thermo Scientific) according to the manufacturer’s instructions. Precleared samples were incubated with 20C3 MAb-bound beads overnight at 4°C with rotation. Beads were washed twice with RIPA buffer before elution in Laemmli sample buffer. 20C3 MAb, lysate input, bead flowthrough, RIPA washes, and bead eluate were resolved on a 4% to 15% polyacrylamide gel (Bio-Rad) and stained with Coomassie blue. Bands in the eluate lanes were excised and analyzed by mass spectrometry.

### Mass spectrometry (MS) analysis.

Excised gel bands were reduced, alkylated, and digested with trypsin. LC-ESI-MS/MS (liquid chromatography-electrospray ionization-tandem mass spectrometry) analysis of the peptide digests was done by C_18_ reversed-phase (RP) chromatography using an Ultimate 3000 RSLCnano system (Thermo Scientific, USA) equipped with an Acclaim PepMap rapid-separation liquid chromatography (RSLC) C_18_ column (Thermo Scientific, USA) (2-μm pore size, 100 Å, 75 μm by 15 cm). The ultra-high-performance liquid chromatography (UHPLC) instrument was connected to a TriVersa NanoMate nano-electrospray source (Advion, USA) and a linear ion trap LTQ-XL (Thermo Scientific, USA) mass spectrometer with ESI source operated in positive-ionization mode. Automated protein identification was performed by the use of Mascot search engine v. 2.5.1 (Matrix Science) against the ToxoV12_uniprot_20150225 database (27,608 entries) with the following search parameters: trypsin; three missed cleavages; peptide charges of +2 and +3; peptide tolerance of 2.0 Da; MS/MS tolerance of 0.8 Da; carbamidomethylation (Cys) for fixed modifications; deamidation (Asn and Gln) and oxidation (Met) for variable modifications. A decoy database search was also performed to measure the false-discovery rate. Mascot protein identification results were further analyzed by the use of Scaffold software v. 4.4.5 based on 99% protein and 95% peptide probabilities.

### Immunofluorescence assay (IFA).

Pru Δ*ku80* Δ*hxgprt* or transgenic parasite strains were inoculated into HFF cells on coverslips and incubated in pH 7 media for 1 day for tachyzoite staining or were differentiated to bradyzoites. Infected coverslips were rinsed twice with phosphate-buffered saline (PBS) before fixation was performed with 4% paraformaldehyde for 20 min followed by permeabilization in 0.2% Tween containing 0.1% glycine for 20 min to quench the fixative. Fixed and permeabilized cells were washed three times with PBS before blocking was performed with 1% bovine serum albumin (BSA)–PBS at room temperature for 1 h. 20C3 MAb, TgME49_209755 PAb, fluorescein isothiocyanate (FITC)-conjugated Dolichos biflorus agglutinin (DBA) (Vector Lab), ALD1 (Kentaro Kato), and SalmonE (7) were used as primary antibodies. Anti-mouse or anti-rabbit goat antibodies conjugated with Alexa Fluor 488 or 594 were used as secondary antibodies. All antibodies were diluted 1:500 in 1% BSA and incubated for 60 min at 37°C followed by three washes with PBS. Coverslips were mounted with ProLong Gold with DAPI (4′,6-diamidino-2-phenylindole; Life Technologies), and images were acquired using a Leica TCS SP5 confocal microscope.

### Polyclonal antibody production.

The N-terminal coding region of MAG2 (aa 284 to 407) was PCR amplified using Q5 High-Fidelity DNA polymerase (New England Biolabs) and MAG2 N-terminal specific primers and ligated into pET32 vector. The resulting vector was transformed into BL21(DE3) competent Escherichia coli (NEB). Transformed bacteria were grown in Luria broth (LB; Sigma-Aldrich) containing ampicillin (100 μg/μl) for 5 to 6 h at 37°C and were induced with isopropyl β-d-1-thiogalactopyranoside (0.1 mM) for protein expression. The bacterial cells were pelleted, resuspended, and sonicated in lysis buffer (1% Triton X-100–10 mM imidazole–7 mM beta-mercaptoethanol–PBS), and the resulting supernatant was purified with nickel beads. Purified peptides were emulsified with Titer Max Gold and injected intraperitoneally into BALB/c^Δdm1^ mice. Immunized mice were boosted every 14 days before sera were collected 1 month after initial immunization and checked for reactivity against *in vitro* T. gondii cysts by IFA. All primers used are listed in Table 2.

**TABLE 2:** Primers

Name	Sequence^*a*^	Purpose
MAG2_Nterm_FWD	**GGTACCGATGACGACGACAAG**GCGACCAAATCCACTTTGA	Peptide expression
MAG2_Nterm_RVS	**GGAGAAGCCCGGGC**TCTCCATAGAACCAGCAGCGG	Peptide expression
MAG2_BirA-TAG_FWD	**TGATTACGCCAAGCTCGGAA**GGCACTGCTGAAGAGCGACT	C-terminal tagging
MAG2_BirA-TAG_RVS	**GGCACGGTGTTGTCCTTGTC**TATGTGTTCCATACGGCGCA	C-terminal tagging
sgMAG2_KO	**GTGCGGCGACCGCACCAGCG**GTTTTAGAGCTAGAAATAGC	Knocking out MAG2
sgMAG2_COMP	**GtcaGGCGACCGCtcaAGCG**GTTTTAGAGCTAGAAATAGC	Complementing MAG2-KO
mScarlet_FWD	ATGGTGAGCAAGGGCGAGG	mScarlet C-terminal tagging
mScarlet_RVS	CTTGTACAGCTCGTCCATGCCG	mScarlet C-terminal tagging
MAG1_Cterm_FWD	**TCGAGCTCGGTAATTTAAAT**CGGTAGTCCATCCGCGTTTT	mScarlet C-terminal tagging
MAG1_Cterm_RVS	**CCTCGCCCTTGCTCACCAT**AGCTGCCTGTTCCGCTAAGA	mScarlet C-terminal tagging
MAG2_Cterm_FWD	**GCGCCATTCGCCATTTAAAT**GAGACAAGACGGAAGCGTCA	mScarlet C-terminal tagging
MAG2_Cterm_RVS	**CCTCGCCCTTGCTCACCAT**TATGTGTTCCATACGGCGCA	mScarlet C-terminal tagging
MAG1_3UTR_FWD	**TGGACGAGCTGTACAAGTAA**ACGCATATCCGCACCAGTTT	mScarlet C-terminal tagging
MAG1_3UTR_RVS	**TCTAGAGGATCCATTTAAAT**GAGCACTTGTCCACGTTTGC	mScarlet C-terminal tagging
MAG2_3UTR_FWD	**TGGACGAGCTGTACAAGTAA**CCGTTCGCGGGTAGGTTAAA	mScarlet C-terminal tagging
MAG2_3UTR_RVS	**TCTAGAGGATCCATTTAAA**TTAGCCTACGCTTTTGCGACT	mScarlet C-terminal tagging
CST1_5UTR_FWD	**CGAGCTCGGTAATTTAAAT**GAACAGAAACACCGCGGCAAG	Secreted mScarlet
CST1_5UTR_RVS	**GCCTCGCCCTTGCTCACCAT**CGCACCTTCGCTCCCGTGAA	Secreted mScarlet
Ku80_5UTR_FWD	**GCTCGGTAATTTAAAT**GTCCTGCGAGGCCTTCAATAG	Secreted mScarlet
Ku80_5UTR_RVS	**TTGCCGCGGTGTTTCTGTTC**AGATAAGATGAAGCCCGTAGGT	Secreted mScarlet
Ku80_3UTR_FWD	**GCAAACGTGGACAAGTGCTC**CTTGGCTAACATCTGCGGGC	Secreted mScarlet
Ku80_3UTR_RVS	**TAGAGGATCCATTTAAAT**TGCCGTGCGTATACATCTATGG	Secreted mScarlet
sgKu80	**GCTTCAGAGAAAAATGCCGTC**GTTTTAGAGCTAGAAATAGC	Secreted mScarlet

a Overhanging regions for Gibson assembly or KLD reaction are indicated in bold. Lowercase sequence characters indicate stop codons targeted by Cas9.

### Endogenous knockout, complementation, and tagging strategy using CRISPR-Cas9.

To knock out MAG2, p-HXGPRT-Cas9-GFP containing a single guide RNA (sgRNA) targeting the N terminus of MAG2 was prepared as previously described (55). Pru Δ*ku80* Δ*hxgprt* or ME49 Δ*hxgprt* Δ*ku80* parasites were cotransfected with 20 μg of uncut circular p-HXGPRT-Cas9-GFP and 280 pmol of each unannealed donor oligonucleotides in incomplete cytomix (56). Donor DNA consisted of 100 bp of oligonucleotides (Thermo Fisher) of the forward and reverse sequences of the N terminus of MAG2 containing four in frame stop codons. After transfection, transgenic parasites were selected with 25 μg/ml mycophenolic acid and 50 μg/ml xanthine (MPA-XA) for 10 days followed by immediate subcloning. Clones of MAG2-negative (ΔMAG2) parasites were screened by IFA for loss of 20C3 MAb signal. Because circular p-HXGPRT-Cas9-GFP was transfected into the parasites, sensitivity to MPA-XA was restored after loss of the HXGPRT resistance cassette after multiple rounds of parasite replication. In addition, loss of pHXGPRT-Cas9-GFP prevents constitutively expressed Cas9-mediated toxicity (57, 58).

To generate the MAG2 complement (MAG2-COMP) parasite line, a different sgRNA targeting the N terminus of MAG2 was ligated into p-HXGPRT-Cas9-GFP. The resulting plasmid was cotransfected with donor oligonucleotides, reversing the stop codons to synonymous mutations into ΔMAG2 parasites by the use of the same transfection and selection method as that described above. Loss of the HXGPRT resistance cassette was confirmed when the negative-control parasites (ΔMAG2 parasites transfected without p-HXGPRT-Cas9-GFP) did not grow under conditions of selection. Sanger sequencing of the N terminus of MAG2 and IFA were used to confirm successful knocking out and complementation of the MAG2 endogenous locus.

To tag MAG1 and MAG2 endogenously with mScarlet, the mScarlet coding sequence was amplified and ligated between bp 500 and 900 of the C terminus and 3’ untranslated region (3’UTR) of the respective genes with pUC19 as the backbone. Amplified donor plasmids were cut with SwaI and cotransfected with sgRNA targeting the C terminus of MAG1 or MAG2 on p-HXGPRT-Cas9-GFP into Pru Δ*ku80* Δ*hxgprt* parasites. Transfected parasites were selected with MPA-XA for 10 days and subcloned by screening for mScarlet fluorescent parasites. All primers and oligonucleotides used are listed in Table 2 and Table 3, respectively.

**TABLE 3:** Oligonucleotides used as donor DNA to generate ΔMAG2 and MAG2-COMP strains

Name	Sequence^*a*^
MAG2_KO_FWD	GCAGAATGAAGCTCTTCTTC**tag**CTGGTGCTC**taa**GGGGTCTCCTCGATCTTTGCTGCCCAGTGCCTCGCT**tga**GCGGTCGCC**tga**C GTGCGGGAATGCC
MAG2_KO_RVS	GGCATTCCCGCACG**tca**GGCGACCGC**tca**AGCGAGGCACTGGGCAGCAAAGATCGAGGAGACCCC**tta**GAGCACCAG**cta**GAAG AAGAGCTTCATTCTGC
MAG2_COMP_FWD	GCAGAATGAAGCTCTTCTTC**AAA**CTGGTGCTC**GCCGGC**GTCTCCTCGATCTTTGCTGCCCAG**TGTTTG**GCT**GGC**GCGGTC**GCC** GCCCGTGCGGGAATGCC
MAG2_COMP_RVS	GGCATTCCCGCACG**GGC**GGCGACCGC**GCC**AGC**CAAACA**CTGGGCAGCAAAGATCGAGGAGAC**GCCGGC**GAGCACCAG**TTT** GAAGAAGAGCTTCATTCTGC

a Mutations are indicated in bold, with stop codons indicated as bold lowercase letters.

### Expression of transgenes.

The coding sequence of MAG2 and ∼2,400 bp upstream of its start codon were PCR amplified using Q5 High-Fidelity DNA polymerase (New England Biolabs) and placed into the pLIC-DHFR-BirA*-3×HA plasmid (19) with the BirA* tag attached to the C terminus of MAG2. Sanger sequencing of this plasmid revealed a truncated MAG2 sequence attached to BirA*-3×HA. The resulting plasmid was cut with PsiI and transfected into the Pru Δ*ku80* Δ*hxgprt* strain for random integration into the genome. Transfected parasites were selected with 1 μM pyrimethamine.

The CST1 promoter-driven mScarlet plasmid was constructed by placing the 5′UTR and signal peptide of CST1 before the N-terminal coding sequence of mScarlet followed by the 3′UTR of MAG1. This transgene was then ligated between (approximately) bp 500 and 750 of the 5′UTR and 3′UTR of the Ku80 gene (17) for direct integration of the mScarlet construct into the Ku80 locus. The CST1 promoter-driven mScarlet plasmid was cut with SwaI and cotransfected with p-HXGPRT-Cas9-GFP containing a sgRNA targeting the Ku80 locus into the Pru Δ*ku80* Δ*hxgprt* strain, and the transfected parasites were selected with MPA-XA. All primers used are listed in Table 2.

### Immunoblotting.

HFF cells that had been infected with T. gondii bradyzoites induced by 3 days of differentiation were harvested, centrifuged at 10,000 × *g*, and lysed in Laemmli buffer. The samples were resolved on a 4% to 15% polyacrylamide gel and transferred to a polyvinylidene difluoride (PVDF) membrane (Millipore). The membrane was blocked with 5% BSA–Tris-buffered saline with 0.1% Tween 20 (TBST) for 1 h at room temperature before being probed with 20C3 MAb or anti-HA rat monoclonal antibody conjugated with horseradish peroxidase (HRP; Roche) at 1:500 for 1 h at room temperature. Following 20C3 MAb primary antibody incubation, the membrane was washed three times with TBST and then incubated with sheep anti-mouse antibody conjugated with HRP (GE Healthcare) for 1 h at room temperature. The membrane was then washed and scanned using an Odyssey imaging system (Li-COR).

### Fluorescence recovery after photobleaching.

Parasites expressing mScarlet were inoculated into HFFs grown on a glass-bottom dish and either analyzed the next day for tachyzoite experiments or differentiated to bradyzoites for 4 days prior to analysis. Infected cells were viewed under a Zeiss LSM 5 Live duo-scan laser scanning microscope at a controlled temperature of 37°C. Images were acquired and analyzed using ZEN 2009 software. The sequence of the experiments consisted of an acquisition step of three images of unbleached vacuoles followed by 10 bleaching steps performed with a 100% laser setting at wavelengths 488 nm and 561 nm; fluorescence recovery images were taken every 2 s for 1 min. A total of 21 cysts/vacuoles were analyzed for each experimental group.

### Cell fractionation.

HFFs were infected and incubated with tachyzoites for 2 days or induced under bradyzoite induction conditions for 3 days. The infected cells were washed and scraped with cold PBS (containing 1× Roche cOmplete protease inhibitor cocktail [with EDTA], 5 mM NaF, and 2 mM activated Na_3_VO_4_). Cells were lysed by passage through 27.5-gauge needles, and intact parasites were separated by low-speed centrifugation at 2,500 × *g* for 10 min at 4°C. The resulting low-speed pellet (LSP) containing intact parasites was saved for further analysis, while the low-speed supernatant (LSS), which contained cyst or parasitophorous vacuole components, was separated into soluble and membrane-associated fractions by high-speed centrifugation at 100,000 × *g* for 1.5 h. The resulting high-speed supernatant (HSS) containing soluble cyst or parasitophorous vacuole components was saved, while the pellet was treated by resuspension in either PBS or 1 M NaCl to free peripheral membrane-associated proteins. The resuspended fractions were centrifuged at 100,000 × *g* to separate salt-released peripheral membrane proteins in the high-speed supernatant (HSS) from the membrane-bound proteins in the high-speed pellet (HSP). Before analysis by immunoblotting was performed, all soluble fractions were concentrated by acetone precipitation.

### Plaque assay.

Fifty purified tachyzoites of each transgenic parasite were inoculated into triplicate wells in 6-well culture plates containing confluent host HFF cells. After 2 weeks of culture without disturbance, cells were fixed with a 20% methanol and 0.5% crystal violet solution for 30 min at room temperature. After fixation, cells were washed once with distilled water before being imaged (Alpha Innotech) and analyzed with ImageJ to measure the size of plaques.

### *In vivo* murine infection for survival curve, cyst number, and cyst size quantification.

C57BL/6 mice (Jackson) were injected intraperitoneally with 1,000 parasites of the Pru Δ*ku80* Δ*hxgprt*, ΔMAG2, or MAG2-COMP strains. Infected mice were monitored for 30 days and noted for death. After 30 days, brains were retrieved from mice that survived and were homogenized in PBS using a Wheaton Potter-Elvehjem tissue grinder (Thermo Fisher) (100-μm to 150-μm clearance). An aliquot of the brain homogenate was screened for GFP fluorescent cysts using a Microphoto-FXA epifluorescence microscope (Nikon). Images of these cysts were analyzed with ImageJ to determine their sizes.

### Transmission electron microscopy (TEM) and immunoelectron microscopy (IEM).

For ultrastructural analyses using a TEM, ME49 Δ*hxgprt* Δ*ku80* and ME49 Δ*hxgprt* Δ*ku80* Δ*mag2* parasites were injected into BALB/c^Δdm1^ mice at 1,000 parasites per mouse, followed by cyst harvesting and purification 3 weeks postinfection. For the Pru Δ*ku80* Δ*hxgprt*, ΔMAG2, and MAG2-COMP strains, cysts were prepared *in vitro* by incubation under bradyzoite induction conditions for 7 days. Cysts were fixed with 2.5% glutaraldehyde–2% paraformaldehyde–0.1 M sodium cacodylate buffer, postfixed with 1% osmium tetroxide followed by 2% uranyl acetate, dehydrated through a graded ethanol series, and embedded in LX112 resin (Ladd Research Industries, Burlington, VT). Ultrathin sections were cut on a Leica Ultracut UC7 ultramicrotome, stained with uranyl acetate followed by lead citrate, and viewed on a JEOL 1400EX transmission electron microscope at 80 kV.

For IEM, ME49 parasites were injected into BALB/c^Δdm1^ mice at 1,000 parasites per mouse, followed by cyst harvesting and purification 3 weeks postinfection. Purified cysts were fixed with 4% paraformaldehyde–0.05% glutaraldehyde–0.1 M sodium cacodylate buffer, dehydrated through a graded ethanol series, with a progressive lowering of the temperature to −50°C in a Leica EMAFS (electron microscopy automatic free substitution) system, embedded in Lowicryl HM-20 monostep resin (Electron Microscopy Sciences), and then polymerized using UV light. Thin sections were immunolabeled with or without 20C3 MAb and were then stained with uranyl acetate (Electron Microscopy Sciences). Stained sections were viewed on a JEOL 1400EX transmission electron microscope at 80 kV.

### Statistics.

Analysis of the mScarlet and GFP recovery curves was performed by fitting the mScarlet and GFP recovery values at each time point over all samples with a linear mixed-effects model using the R package lme4. *Post hoc* comparisons were performed to determine the time points that represented significant differences between mScarlet and GFP recovery percentages by the use of the R package emmeans. For analysis of the FRAP parameters obtained from the ZEN 2009 program, the Welch two-sample *t* test was used to determine whether there were significant differences in the immobile fractions or in the time of equilibrium between mScarlet and GFP.

Data obtained from counting cysts and determining cyst sizes were graphed in R as violin plots. To compare multiple groups, one-way analysis of variance (ANOVA) followed by a *post hoc* Tukey honestly significant difference (HSD) test was performed. Comparisons of survival curves for mice infected with different parasite strains were performed using the R packages survminer and survival. The statistical significance of the survival curves was determined by the Gehan-Wilcox test. Pairwise comparisons of mice infected by each strain were performed using the Peto & Peto test.
