# Supplementary figures and images for: MAG2, a Toxoplasma gondii Bradyzoite Stage-Specific Cyst Matrix Protein
Source: mSphere. 2020 Feb 19;5(1):e00100-20. doi: 10.1128/mSphere.00100-20 (PMC7031614; doi:10.1128/mSphere.00100-20)

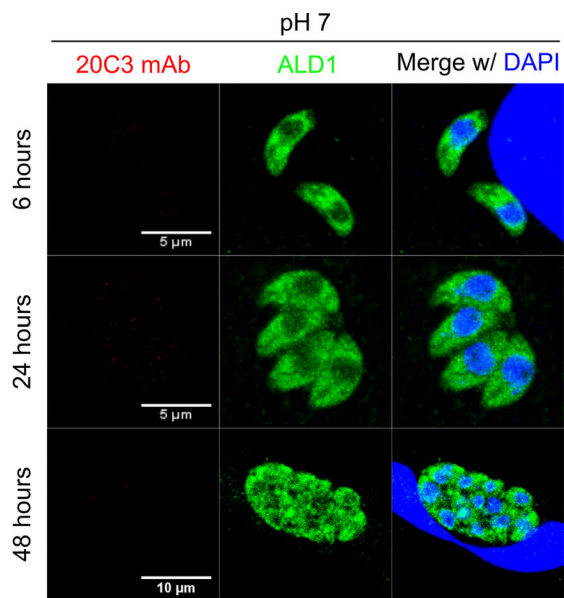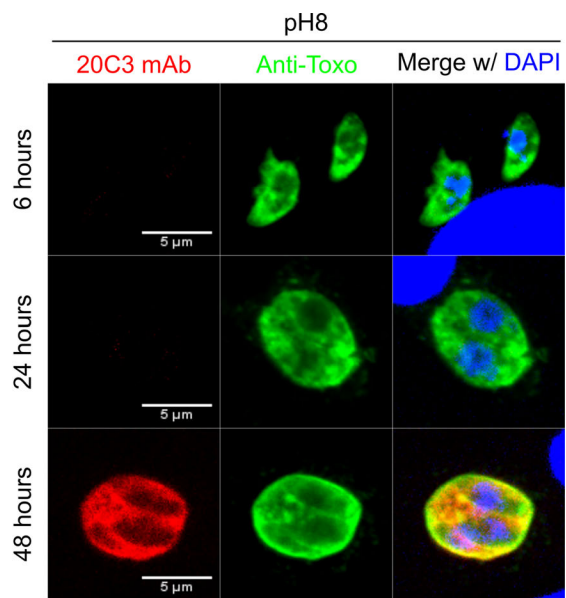

Supplement: FIG S1 [file mSphere.00100-20-sf001.pdf]

A

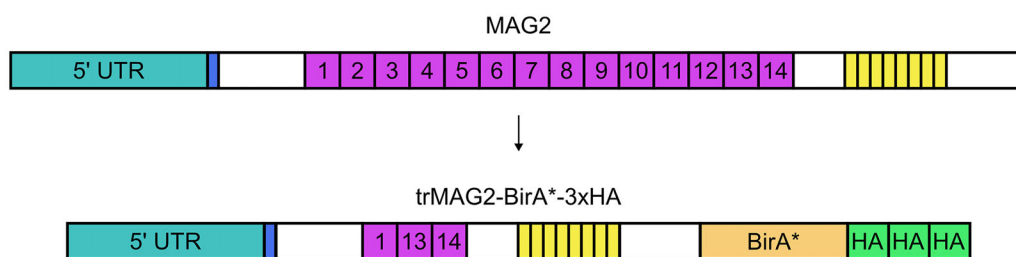

B

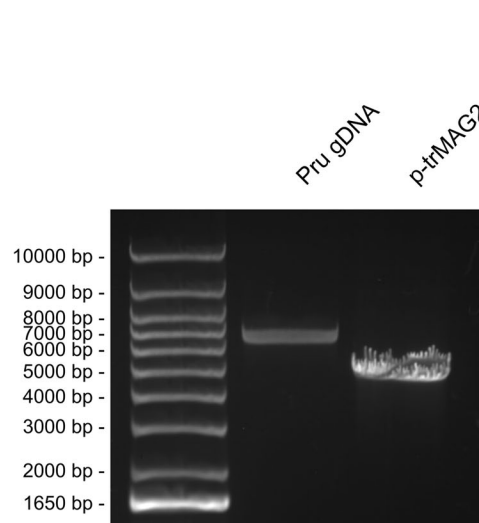

C

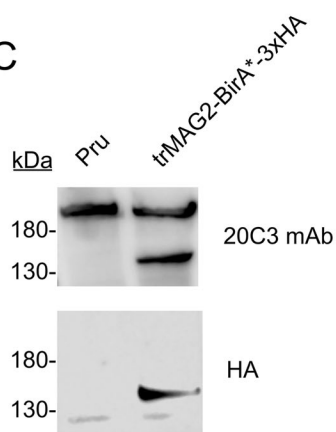

D

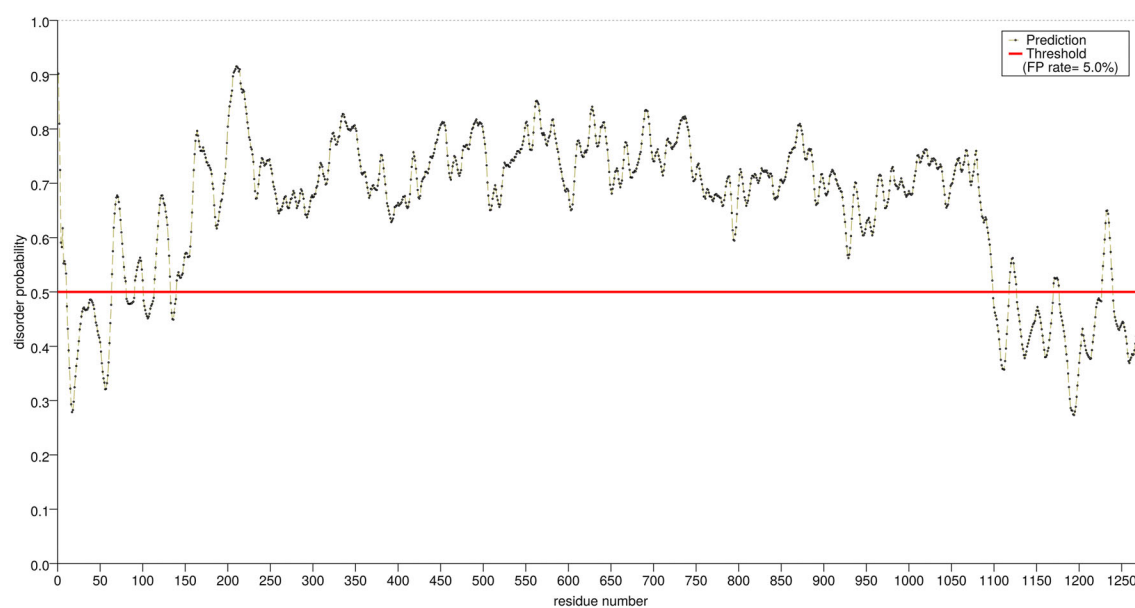

Supplement: FIG S2 [file mSphere.00100-20-sf002.pdf]

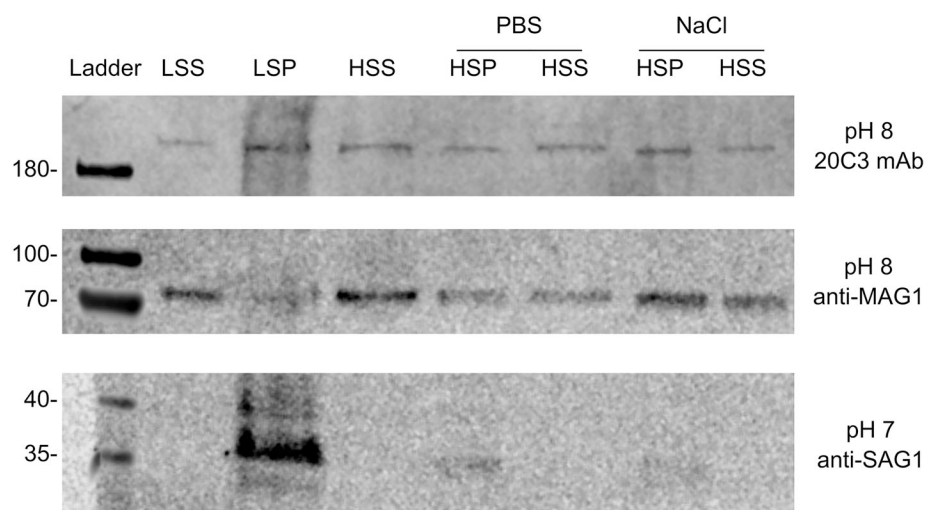

Supplement: FIG S3 [file mSphere.00100-20-sf003.pdf]

A

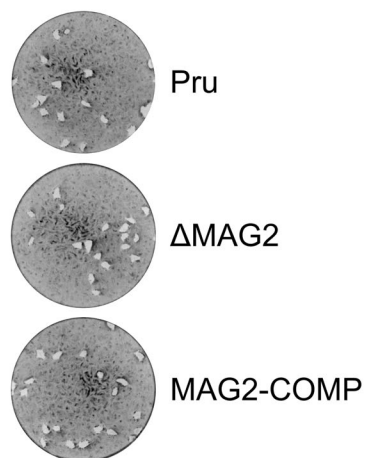

B

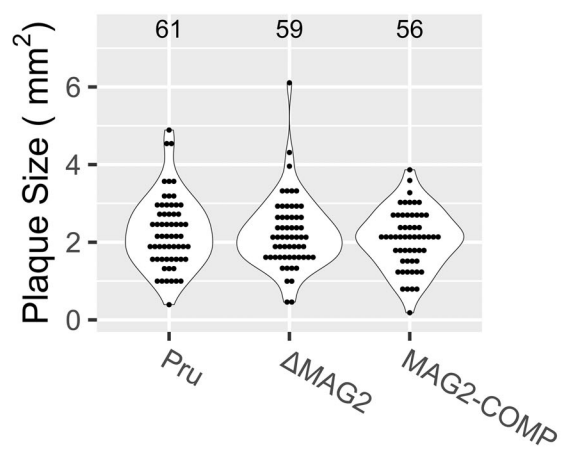

C

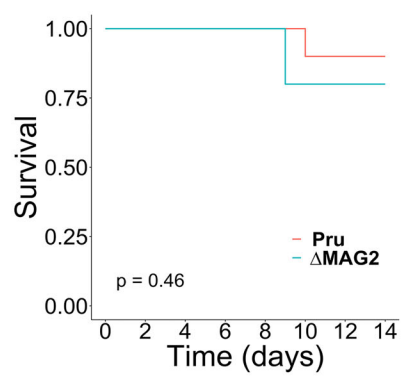

Supplement: FIG S4 [file mSphere.00100-20-sf004.pdf]

A

Pru

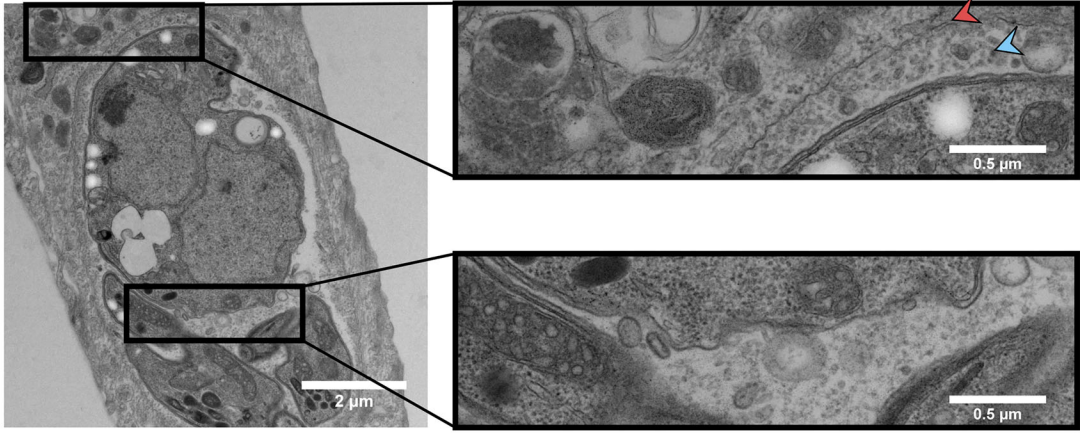

$\Delta$ MAG2

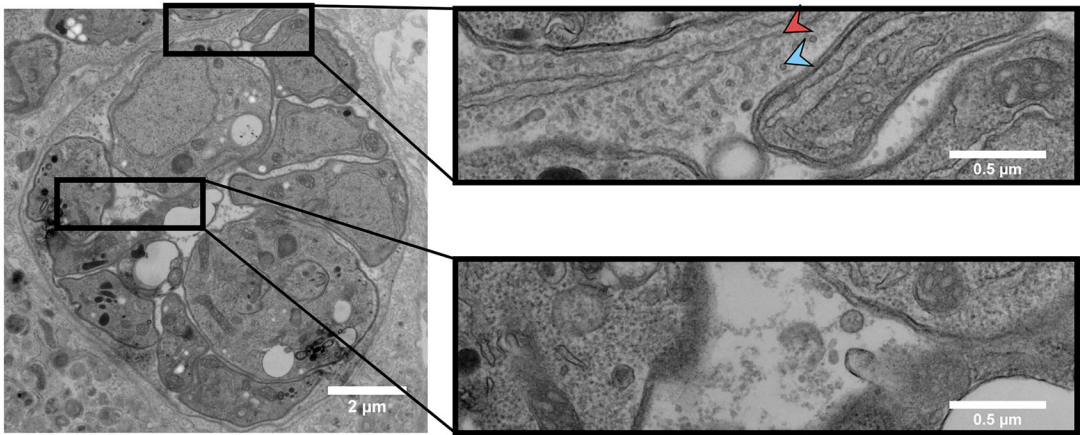

MAG2-COMP

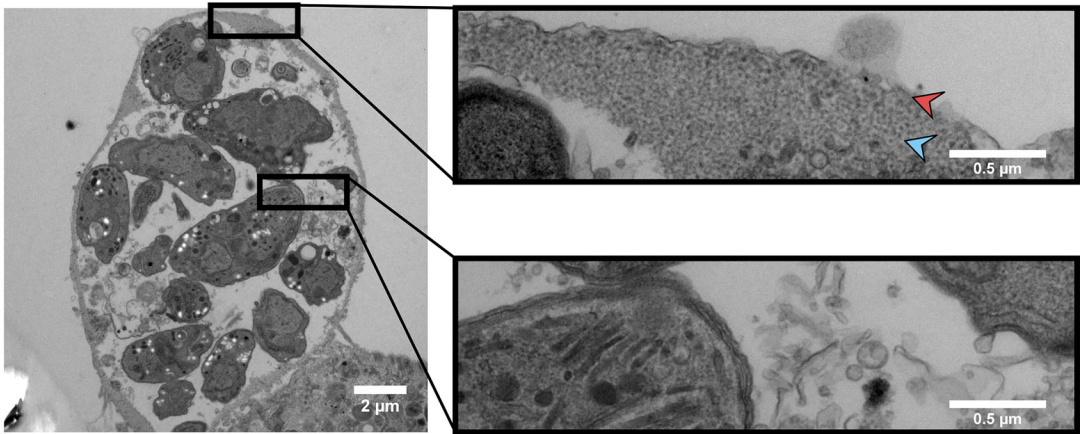

Supplement: FIG S5 [file mSphere.00100-20-sf005.pdf]
